# Evaluation of efficacy and safety of percutaneous transforaminal endoscopic surgery (PTES) for surgical treatment of calcified lumbar disc herniation: a retrospective cohort study of 101 patients

**DOI:** 10.1186/s12891-020-03938-3

**Published:** 2021-01-12

**Authors:** Hao Wang, Tianyao Zhou, Yutong Gu, Zuoqin Yan

**Affiliations:** 1 Department of Orthopaedic Surgery, Zhongshan Hospital Fudan University, 200032 Shanghai, China; 2 Shanghai Medical College, Fudan University, 200032 Shanghai, China; 3 Department of Orthopaedic Surgery, Shanghai Public Health Clinical Center, Fudan University, 201508 Shanghai, China

**Keywords:** Calcified lumbar disc herniation, Transforaminal, Endoscopic discectomy, Minimally invasive surgery

## Abstract

**Background:**

Percutaneous transforaminal endoscopy has been widely used to treat lumbar disc herniation (LDH), but the steep learning curve and difficulties in removing the calcified disc hinders the application of conventional endoscopy in treating calcified lumbar disc herniation (CLDH). In 2017, we first reported Percutaneous Transforaminal Endoscopic Surgery (PTES) as an easy-to-learn posterolateral transforaminal endoscopic technique to decompress the nerve root for LDH. We used our PTES technique to remove the calcified LDH and the purpose of this study is to evaluate the safety and efficacy of this technique.

**Methods:**

Forty-six patients with CLDH and fifty-five patients with uncalcified lumbar disc herniation (ULDH) underwent PTES to decompress the nerve root. Visual analogue scale was collected before the surgery, immediately, one week, one month, two months, three months, six months, 12 months and 24 months after surgery. The outcomes of MacNab classification were collected 24 months after surgery. Intra- and Post-operative complications were also recorded.

**Results:**

For CLDH patients, the VAS score was 9 (5–10) before operation, and then dropped to 2 (1–4) after surgery. VAS score continually decreased to 0 (0–3) at 24 months after surgery. 95.65% of CLDH patients showed excellent or good outcomes. ULDH group showed similar MacNab classification (94.55%) and VAS changing curve. The therapeutic effect of PTES in treating CLDH was as good as that in treating uncalcified patients.

**Conclusions:**

PTES is an effective and safe method to treat calcified lumbar disc herniation.

## Background

Lumbar disc herniation (LDH) is one of the commonest intervertebral disc degenerative diseases. Herniated disc compresses and irritates the nerve root, resulting in radiating leg pain [1]. More than 80% of the patients could respond to conservative patients including bed rest and oral analgesics, but for selective patients, surgical discectomy leads to a faster relief [2].

Calcified lumbar disc herniation (CLDH) is a subtype of LDH with the herniated site calcified. Existing studies blamed calcification on the longer course of disease, application of Traditional Chinese Medicine, developmental changes in nucleus pulposus and unknown triggering factors such as infection and microtrauma [3–5]. The adhesion between the calcification and nerve root or dura mater increases the surgical difficulty and may cause iatrogenic injury such as nerve root injury and dural tear [3, 5].

Transforaminal endoscopy has certain advantages including local anesthesia, small trauma, fast recovery and precisive discectomy. This technique has been proven to be safe and effective to treat ordinary LDH [3, 6, 7]. But for CLDH, conventional endoscopic techniques have difficulty to remove the calcification. The removal procedure could be challenging and may well result in iatrogenic damage [3]. Until now, laminectomy and discectomy through open-surgery are still the commonest therapeutic plan.

Percutaneous Transforaminal Endoscopic Surgery (PTES), our self-created transforaminal endoscopic technique, has the advantages of simple orientation, easy puncture, reduced steps and less fluoroscopic X-ray exposure over conventional endoscopic techniques. It has been shown to treat various types of LDH safely and efficiently, including CLDH, but limited sample size and lack of control group in our previous study made the outcome less convincing [8]. In this study, we treated and followed up more patients to evaluate the safety and efficacy of PTES in dealing with CLDH.

## Methods

### Data collection

This retrospective cohort study was approved by the Ethics Committee of Zhongshan Hospital. We collected the data of consecutive hospitalized patients of LDH treated with PTES in our hospital between January 2015 and December 2017. 101 patients who underwent PTES and met the following requirements were included in this study: (1) Patients complained of primarily radicular pain of unilateral leg; (2) Clear nerve root compression sign including positive *Lasegue* sign, sensory or movement disorder of the lower limbs and the reflex abnormalities of knee or ankle; (3) Imaging data confirmed the presence of single-level LDH and excluded other spinal diseases such as lumbar spondylolisthesis or lateral recess stenosis; (4) Conservative treatment failed.

**Fig. 1:**
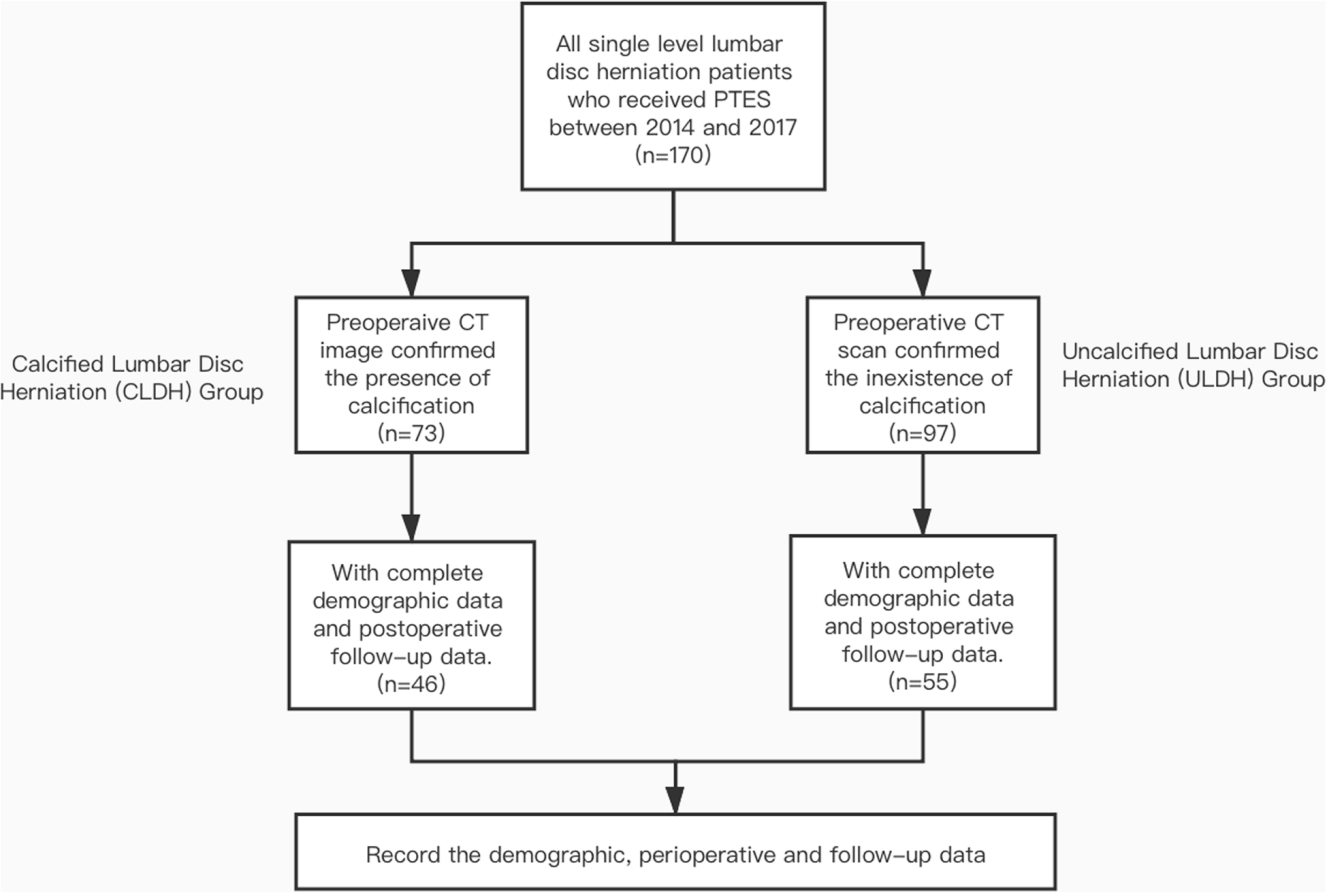
Flowchart demonstrating the grouping criteria of this study

In these patients, preoperative CT scan image confirmed that 46 of them had calcification at the herniated site. Patients with calcified herniation were included in CLDH group and the rest of them were included in ULDH (Uncalcified Lumbar Disc Herniation) group. The detailed grouping criteria is shown in Fig. 1.

Table 1 summarizes the baseline data of two groups. The CLDH group was comprised of 46 patients and ULDH group included 55 patients. There was no statistical significance in age, gender, BMI, operation segment and follow-up time between two groups.

**Table 1:** The demographic data of CLDH and ULDH patients

		CLDH (*n* = 46)	ULDH (*n* = 55)	*p*-value
Age		49.57 ± 16.17	51.51 ± 16.07	0.547 ^a^
Sex	M	26	31	0.987 ^b^
	F	20	24	
BMI		24.38 ± 3.43	24.84 ± 3.15	0.482 ^a^
Segments	L4-L5	26	36	0.358 ^b^
	L5-S1	20	19	
Follow-up time (month)		24 (24–29)	24 (24–28)	0.285 ^c^

^a^ Exhibited in the format of “Mean ± standard deviation” and tested by Student’s t test

^b^ Pearson’s chi-squared test

^c^ Exhibited in the format of “Median (Min-Max)” and tested by Mann-Whitney U test

### Surgical techniques

All the surgeries were undertaken by the same senior surgeon. The patient was placed in a prone position on a radiolucent table with conscious sedation and C-arm was used for intraoperative fluoroscopic imaging.

**Fig. 2:**
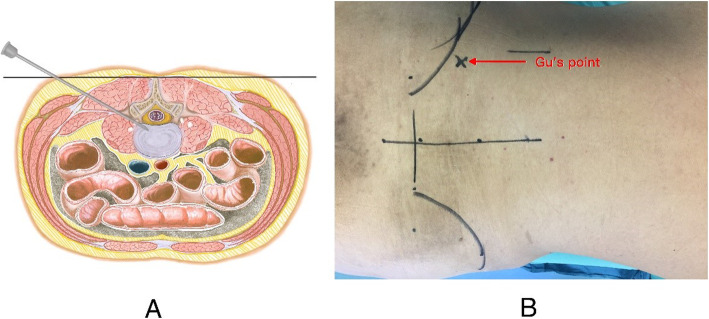
Puncture procedure of PTES. **a** Schematic diagram of the puncture. The entrance point locates at the corner of flat back turning to lateral side at the height of target disc or cranially or slightly caudally. After local anesthesia, an 18-gauge puncture needle was inserted at an angle of about 45° (25°-75°) until reaching the posterior 1/3 of the intervertebral space or intracanal area close to posterior wall of the disc on lateral view and near the medial border of the pedicle on posteroanterior view. **b** The entrance point on real body surface

**Fig. 3:**
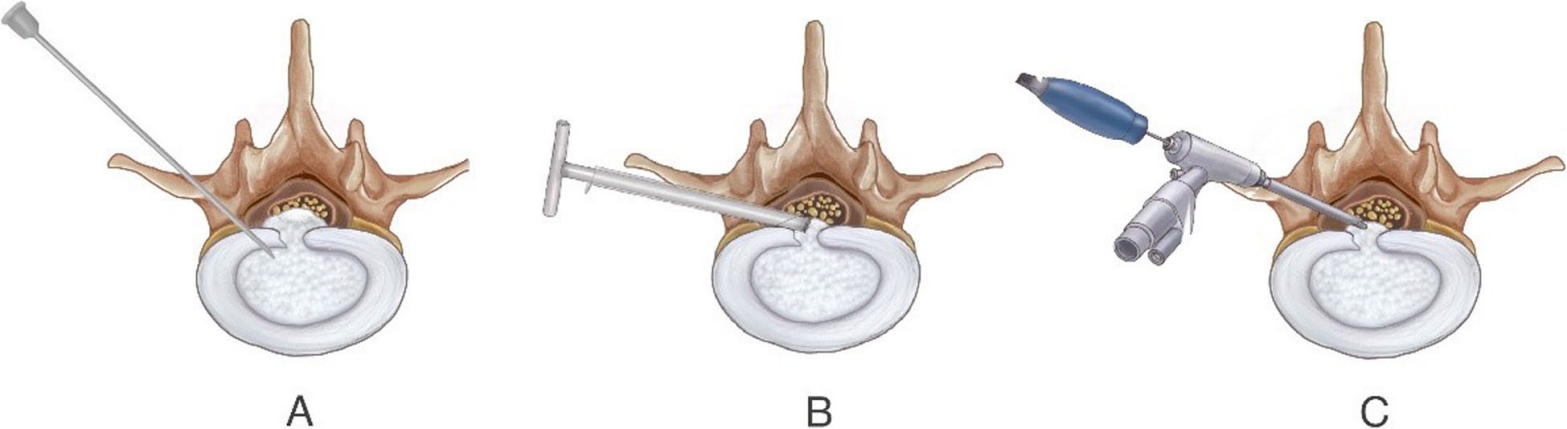
Schematic diagram of procedures of removing the calcified herniation. **a** Insert an 18-gauge puncture needle to the posterior 1/3 of the intervertebral space. **b** Press-down Enlargement of Foramen: through an 8.8-mm diameter cannula, use a 7.5 mm trephine to enlarge the foramen and simultaneously remove the calcified herniation. Compared with removing the uncalcified herniation, the trephine was pressed down further and deepened to remove the calcification directly. **c** Most of the calcification was removed. Rest of the calcification was removed using trephine, electric drill or ultrasonic bone scalpel under endoscopic view

**Fig. 4:**
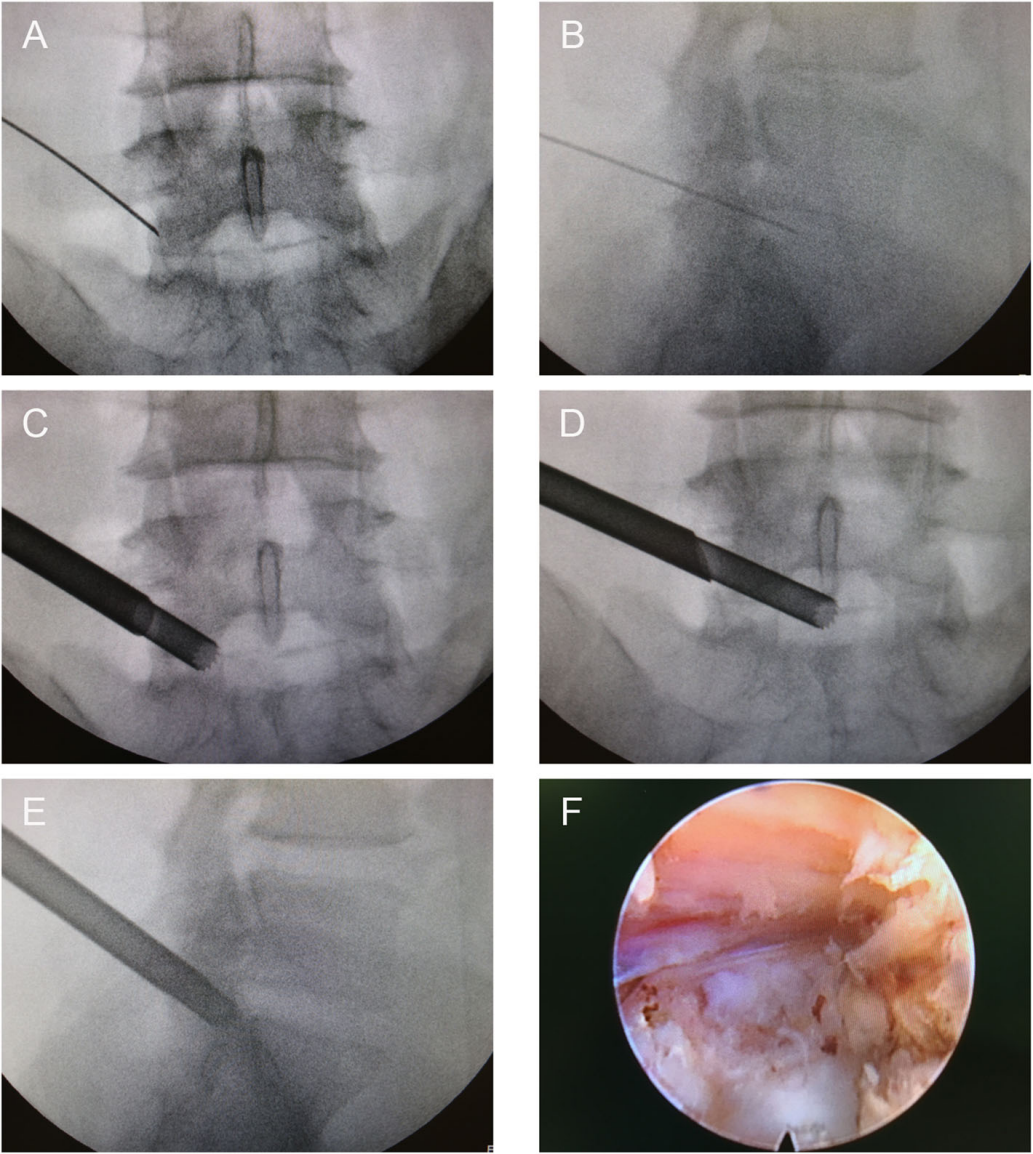
Intraoperative X-ray and endoscopic images of the surgery. **a** Posteroanterior X-ray image of the puncture procedure. **b **Lateral X-ray image of the puncture procedure. **c** Drill the trephine until the distal end reaches the medial border of the pedicle on posteroanterior view. **d**, **e** Press down the trephine further and keep drilling until the distal end exceeds the midpoint between medial border of the pedicle and the spinous process on posteroanterior (**d**) and lateral (**e**) view. **f** Endoscopic view after removing the herniation

The surface marking of anatomic disc space is a transverse line drawn along the metal rod which is placed transversely across the center of the target disc on the posteroanterior image. The entrance point locates at the corner of flat back turning to lateral side at the height of target disc, or cranially or slightly caudally. This entrance point, named “Gu’s point” was easy to determine without the fluoroscopy regardless of different age, gender and body size (Fig. 2). The intersection of posterior midline and the transverse line was the aiming reference point. After local infiltration anesthesia with 1% lidocaine at the entrance point, an 18-gauge puncture needle was inserted anteromedially at an angle of about 45° (25°-75°, adjust based on the actual situation) to horizontal plane. Once the resistance disappeared, the needle tip should stay at posterior 1/3 of the intervertebral space or intracanal area close to posterior wall of the disc on lateral view and near the medial border of the pedicle on posteroanterior view, proving the success of the puncture (Figs. 3a and 4a and b). After dilating the puncture tract stepwise, a 6.3-mm diameter guiding rod was introduced over the guiding wire into the intervertebral foramen and an 8.8-mm diameter cannula with one-side opening was inserted over the guiding rod and docked at the superior facet. Then press down the cannula to decrease the inclination angle and a 7.5-mm diameter trephine was introduced through the cannula to remove the ventral bone of superior articular process to enlarge the foramen. When resistance disappeared, the distal end of the trephine should exceed the medial border of the pedicle on posteroanterior view and reach the posterior wall of disc on lateral view. This enlargement procedure was first introduced by us and was named “Press-Down Enlargement of Foramen” (Figs. 3b and 4c). In order to excise the calcification directly, the trephine was pressed down further to continue drilling until the distal end exceeded the midpoint between medial border of the pedicle and the spinous process on posteroanterior view and was across the posterior wall of disc on lateral view (Fig. 4d and e).

The 7.5-mm diameter working cannula was inserted over the guiding rod. The endoscope was introduced and the herniated tissue could be observed on screen generally. Remove the herniated tissue to free the compressed nerve root. The residual calcified tissue could be removed by small reamer, electric drill or ultrasonic osteotome (Figs. 3c and 4f). The freed nerve root always pulsated in pace with heart beat. After inquiring the patient to confirm the relieved symptoms, the endoscopic surgery could be completed.

Patients were immobilized within five hours after the surgery and left the hospital one day after operation. A flexible brace was used for two weeks. After leaving hospital, patients were encouraged to return to daily life and followed up regularly.

### Pre- and postoperative image

**Fig. 5:**
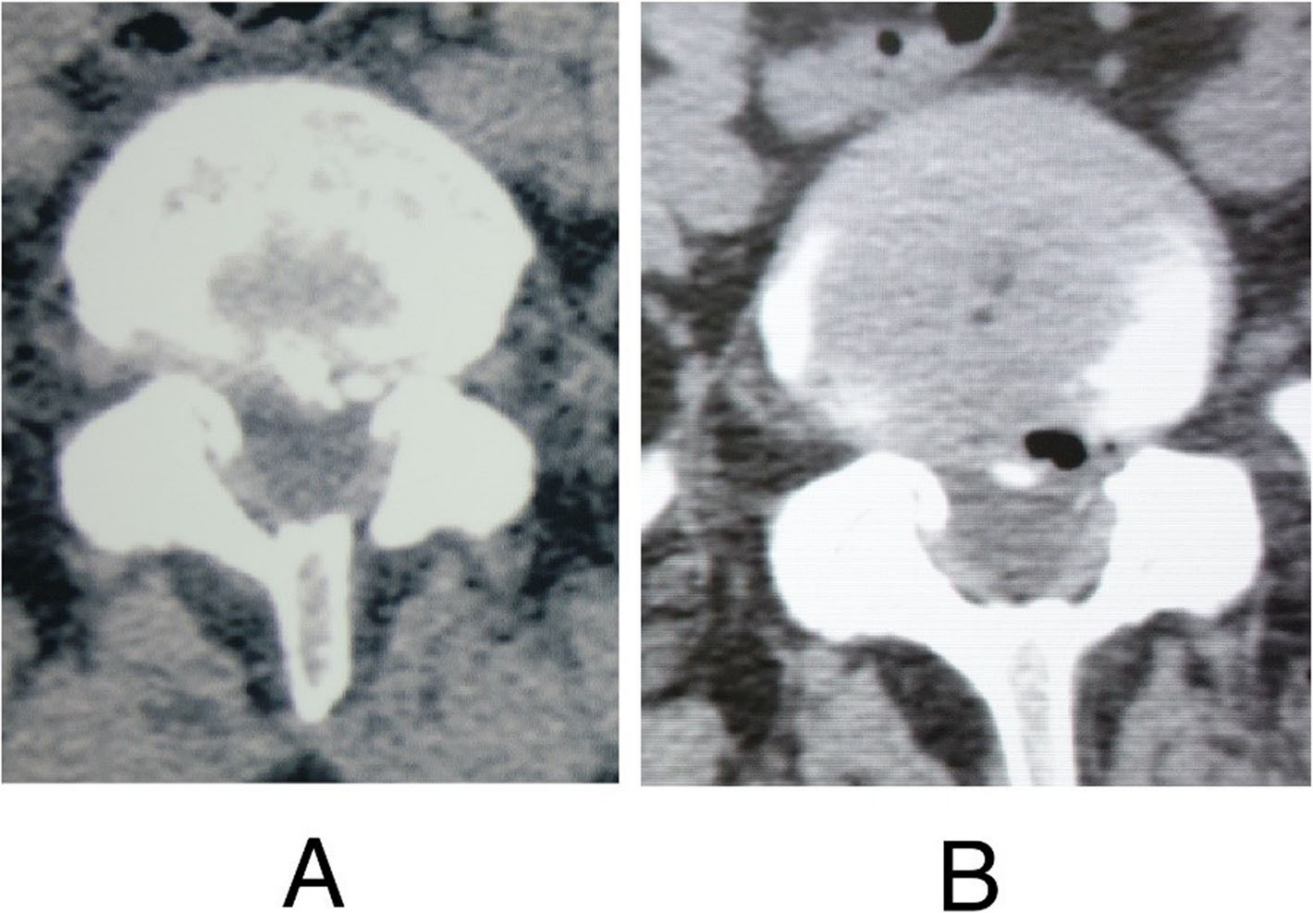
Preoperative and postoperative CT scan images of a 46-year-old female patient who had radiating leg pain for one month and received PTES. .**a** Preoperative CT scan image showed calcified herniation at L5/S1 level. **b** Postoperative CT scan image confirmed the removal of the calcification

Before the surgery, patients received MRI to determine the herniated level and CT scan to confirm the presence of calcification. Posteroanterior and lateral X-rays were required to detect scoliosis or high iliac crest when the lower plate of L4 vertebral body was not higher than bilateral iliac crest. After operation, CT scan was undertaken to confirm the excision of calcification (Fig. 5). Postoperative MRI images were acquired to evaluate decompression outcome and exclude hematoma and dural sac rupture or spinal fluid leakage.

### Clinical follow-up

The pain intensity of the lower limbs was graded using Visual Analogue Scale (VAS) pain score before operation, immediately, one week, one month, two months, three months, six months, 12 months and 24 months after surgery. The therapeutic results were graded 24 months after discharge based on the MacNab criteria. Clinical follow-up was carried out through outpatient or telephone follow-up.

During the follow-up, postoperative complications were recorded including infection, increased weakness of quadriceps, foot/toe extensor or triceps strength and residual or the recurrence of the herniation.

### Statistical analysis

Data analysis was performed by SPSS, version 25.0 (IBM Corp., Armonk, New York, USA). Normal distribution of variable was tested by Shapiro-Wilk test. Normal distribution variable comparation between two groups was tested by Student’s t-test. Non-normal distribution variable and ordinal categorical variable was tested using Mann-Whitney U test. Unordered categorical variable was compared through Pearson’s Chi-squared test or Fisher’s exact test. All significance tests were two-tailed and p < 0.05 was considered statistically significant.

## Results

Perioperative data is shown in Table 2. For the CLDH group, the mean duration of the operation was 64.74 ± 11.82 minutes, which was a little bit longer than that of the ULDH group (58.83 ± 15.52 minutes) but without statistical significance. The mean frequency of intraoperative fluoroscopy was 6 (5–16), which was also slightly more than that of the ULDH group (5 (4–14)). The average hospitalization days were 3 (2–4) days and there was no statistical significance compared with ULDH group. The blood loss was 5 (2–20) ml in CLDH group and the data was similar in two groups.

**Table 2:** The perioperative data

	CLDH	ULDH	*p*-value
Duration of operation (min)^a^	64.74 ± 11.82	58.83 ± 15.52	0.087
Frequency of intraoperative fluoroscopy^b^	6 (5–16)	5 (4–14)	0.090
Hospitalization days^b^	3 (2–4)	3 (2–5)	0.777
Blood loss^b^	5 (2–20)	5 (5–25)	0.187

^a^Exhibited in the format of “Mean ± standard deviation” and tested by Student’s t test

^b^Exhibited in the format of “Median (Min-Max)” and tested by Mann-Whitney U test

The pain degree was evaluated using VAS and the data is exhibited in Table 3. The average preoperative VAS score of CLDH group was 9 (5–10) and it decreased to 2 (1–4) immediately and continually dropped to 0 (0–3) 24 months after operation. VAS score of ULDH group dropped from 8 (6–10) to 2 (0–4) immediately and continually dropped to 0 (0–3) 24 months after surgery. There was no statistical difference of the VAS before surgery between two groups, but VAS score of CLDH group was higher than that of the ULDH group immediately after surgery. One patient in CLDH group and two patients in ULDH group showed rebound effect of leg pain. VAS score of these patients rose at 1 week after surgery and got relieved within 2 months. MacNab classification data was shown in Table 4. Of 46 CLDH patients, 44 (95.65%) patients considered the treatment effect as Excellent or Good 24 months after surgery. The Excellent or Good rate of ULDH group was 94.55% and there was no statistical significance between two groups.

**Table 3:** The preoperative and postoperative VAS data

	Preoperative	Immediately	1 week	1 month	2 months	3 months	6 months	12 months	24 months
CLDH	9 (5–10)	2 (1–4)	2 (1–8)	2 (0–5)	1 (0–4)	1 (0–3)	1 (0–3)	1 (0–3)	0 (0–3)
ULDH	8 (6–10)	2 (0–4)	2 (0–9)	2 (0–6)	1 (0–4)	1 (0–3)	1 (0–3)	1 (0–2)	0 (0–3)
*p*-value^a^	0.211	0.049	0.152	0.893	0.641	0.693	0.994	0.520	0.774

^a^ Values are expressed in the format of “Median (Min-Max)”. Mann-Whitney U test was used to compare the VAS score between CLDH and ULDH groups at every point of time

**Table 4:** The MacNab classification data at 24 months after surgery

	CLDH	ULDH
Excellent	39	42
Good	5	10
Fair	2	2
Poor	0	1
Excellent or good rate	95.65%	94.55%
*p*-value^a^	1.000	

^a^ Excellent or good rate is tested by Fisher’s exact test

Intraoperative and postoperative complications were also recorded and exhibited in Table 5. For CLDH group, one patient had nerve root sleeves rupture. And for ULDH group, two patient encountered nerve root sleeves rupture and another one patient encountered herniation recurrence. Patients of nerve root sleeves rupture did not confront cerebrospinal fluid leakage or other abnormal clinical symptoms. The only one recurrence patient received PTES again and showed favorable prognosis. No one had infection, hematoma, increased weakness of quadriceps or foot/toe extensor strength, or nerve root injury.

**Table 5:** Complications of PTES

	Complications	CLDH	ULDH
Intraoperative	Nerve root injury	0	0
	Nerve root sleeves rupture	1	2
	Dural sac rupture or leakage of CSF	0	0
Postoperative	Infection	0	0
	Hematoma	0	0
	Increased weakness of quadriceps or foot/toe extensor strength	0	0
	Rebound of leg pain	1	2
	Residual or recurrence	0	1
Total		2	5

## Discussion

CLDH is a special subtype of disc herniation. Most researches concerning calcified disc concentrated on thoracic and thoracolumbar vertebrae because of the complexity of the surgical procedure and serious consequence if left untreated [9]. The narrower spinal canal limits the manipulating field. Voluminous herniated disc and severe adhesion to thecal sac may result in iatrogenic damage [10]. Beyond that, wide visualization of the lesion is necessary while maintaining the postoperative spinal stability restricts the usage of osteotomy. Traditional laminectomy is not suitable for thoracic and thoracolumbar calcified herniation [11]. But for CLDH attracted less attention because laminectomy and discectomy, TLIF (Transforaminal Lumbar Interbody Fusion) and PLIF (Posterior Lumbar Interbody Fusion) could remove the calcification efficiently and safely. With the prosperity of transforaminal endoscopic surgery, this minimally-invasive, rapid-recovering and cost-saving technique has been widely applied in the treatment of LDH [12, 13]. Is it possible to treat CLDH through transforaminal endoscopy? May it cause severe iatrogenic injury? The feasibility, safety and efficacy of transforaminal endoscopic surgery to treat CLDH should be studied.

We designed a simplified and effective technique of transforaminal endoscopy-PTES [8].Compared with TESS (Transforaminal Endoscopic Spine Surgery) or YESS (Yeung Endoscopic Spine Surgery), only one posteroanterior fluoroscopy is required to determine the surface projection of targeting segment, and the entrance point locates at the corner of flat back turning to lateral side, which was named “Gu’s Point” [6]. It is not necessary to take the C-arm projection and measure the distance lateral from the midline for determination of the entrance point. This entrance point locates at a more medial position than that of other transforaminal endoscopic techniques, which has three advantages: (1) Avoid injuring the exiting nerve root. Exiting nerve root leaves the foraminal in the direction from superomedial to inferolateral. If the entrance point locates laterally, the foraminotomy procedure may meet and injure the exiting nerve root more possibly and the patient may complain of pain in lower extremities during surgery. (2) Avoid blockage by the high iliac crest for the L5/S1 level. Peak of the iliac crest locates at the lateral side of the waist and the height lowers down when getting closer to the midline. Height of the iliac crest at “Gu’s Point” is relatively lower, reducing the difficulty of puncture and subsequent operation. (3) Avoid injuring abdominal viscera and main blood vessels. Puncture from a lateral entrance point could be dangerous if penetrating into the abdomen. Puncture from “Gu’s Point” is much safer, even if in a large horizontal angle. Tip of the needle could be blocked by bony structure of spine.

During the puncture procedure of PTES, tip of the needle is required to stay at the posterior 1/3 of the intervertebral space or the posterior wall of the disc, making the puncture angle more flexible. When enlarging the foramen, pressing down the cannula docking at the superior articular process to decrease the horizontal angle of the trephine could remove more bones in the ventral part of the articular process, and inserting the cannula into the interspace between disc and dura mater will be much easier. This self-created foraminotomy was called “press-down enlargement of foramen”, which makes it possible to remove the herniated nucleus pulposus compressing dura or contralateral nerve root. Of 209 LDH patients who received PTES, 95.7% showed an excellent or good outcome [8]. For the treatment of CLDH, the extent of pressing down is greater than normal to aim the trephine at the calcification during foraminotomy. Keep drilling until the distal end of the trephine exceeds the midpoint between medial border of the pedicle and the spinous process on posteroanterior view to remove the calcification. Pay close attention to patient’s reaction and stop drilling if patient develops nerve root stimulating symptoms. Preoperative images, intraoperative fluoroscopy and patient’s reaction could guarantee the safety of the operation. Rest of the calcified tissue should be grinded off as completely as possible through trephine, electric drill or ultrasonic bone scalpel under endoscopic vision.

In this study, 44 of 46 CLDH patients indicated a satisfied prognosis two years after surgery and postoperative VAS scores had no statistical difference compared with ULDH patients, proving that PTES could treat CLDH safely and efficiently. Duration of operation and intraoperative frequency of fluoroscopy were slightly more than those of the uncalcified patients, because removing the calcification during foraminotomy needed extra fluoroscopic positioning and using trephine or drill under endoscope may cost more time. We ascribed the low recurrence rate to successful postoperative education. Patients were required to avoid bending down, lifting heavy stuff, maintaining a posture for a long time or focusing force on waist while sneezing or coughing. The removed protruded nucleus pulposus under endoscope is usually fragmentized or sequestrated, and the remaining portion at the intervertebral space is healthy and relatively intact. Generally, the remaining nucleus pulposus could keep stable and will not protrude again. If neglecting postoperative waist maintenance, the intact nucleus pulposus may rupture and protrude again.

This research has two limitations. First, the follow-up time is relatively short. If the patients were followed up for over ten years, the recurrence rate will be more persuasive to reflect the long-term therapeutic effect of PTES. Second, the sample size is still limited. So, we cannot perform further analysis on the relationship between the prognosis and the properties of the calcification, such as the size, location, shape and density.

CLDH has a frequent morbidity and can be relatively difficult to treat. Though PTES technique is a simple, safe and efficient therapeutic method, in our subsequent researches, we will seek for the clinical characteristics of CLDH patients, such as certain risk factors, and the difference in age, course of disease, pain and limited mobility with uncalcified patients. Revealing the etiologies and mechanism of herniated disc calcification could help preventing and blocking the procedure in early stage. We are intending to collect the data of all the CLDH and ULDH patients of recent ten years to analyze the risk factors of calcification in subsequent studies.

## Conclusions

PTES is an effective and safe method to treat CLDH with simple orientation, easy puncture, reduced steps and fewer frequency of fluoroscopy.

## Data Availability

All the data and materials concerning this research are available within the article.

## Abbreviations

PTES: Percutaneous Transforaminal Endoscopic Surgery
LDH: Lumbar Disc Herniation
CLDH: Calcified Lumbar Disc Herniation
ULDH: Uncalcified Lumbar Disc Herniation
VAS: Visual Analogue Scale
TLIF: Transforaminal Lumbar Interbody Fusion
PLIF: Posterior Lumbar Interbody Fusion
TESS: Transforaminal Endoscopic Spine Surgery
YESS: Yeung Endoscopic Spine Surgery
